# Graphene oxide-ferrite hybrid framework as enhanced broadband absorption in gigahertz frequencies

**DOI:** 10.1038/s41598-019-48487-5

**Published:** 2019-08-20

**Authors:** Rajarshi Bhattacharyya, Om Prakash, Somnath Roy, Akhilendra Pratap Singh, Tapas Kumar Bhattacharya, Pralay Maiti, Somak Bhattacharyya, Santanu Das

**Affiliations:** 1 Department of Ceramic Engineering, Indian Institute of Technology (Banaras Hindu University), Varanasi, 221005 Uttar Pradesh India; 2 School of Materials Science and Technology, Indian Institute of Technology (Banaras Hindu University), Varanasi, 221005 Uttar Pradesh India; 3 Department of Physics, Institute of Science, Banaras Hindu University, Varanasi, 221005 Uttar Pradesh India; 4 Department of Electronics Engineering, Indian Institute of Technology (Banaras Hindu University), Varanasi, 221005 Uttar Pradesh India; 5 Department of Ceramic Technology, Government College of Engineering and Ceramic Technology, Kolkata, 700010 West Bengal India

**Keywords:** Electronic properties and materials, Metamaterials

## Abstract

The present investigation is focused on the *in-situ* synthesis of Graphene oxide (GO)-ferrite nanoparticle hybrid framework by gel-combustion method followed by fabrication of homogeneous, structurally stable thin (~100–120 μm) hybrid-polyurethane coating on a metallic aluminum substrate and its application on the properties of broadband absorption over the microwave frequency region. Microstructure studies of hybrid materials illustrated that small sized ferrite nanoparticles (~17 nm) are grafted on and through the graphene layers, which forms a homogeneous coating thereby. The hybrid-nanocomposite coating demonstrated superior broadband absorption properties with absorptivity higher than 90% throughout a bandwidth of ~6 GHz, and moreover, it was found that with increased loading of GO in the nanocomposite, the bandwidth range of absorption frequency increases with enhanced absorptivity. The real part and imaginary part of the surface impedance values of the coating was obtained as 377 Ω and 0 Ω, respectively, which imply that the free-space impedance of the hybrid-nanocomposite coating is matching correctly. The nanocomposite coating showed ultra-high absorptivity over the frequency band of 8–12 GHz, which has numerous practical applications as radar absorbing materials (RAM), stealth technology, electromagnetic shielding, and radiated electromagnetic interference (EMI) management in onboard spacecraft and many more.

## Introduction

The rapid advances in computer technology, communication devices, and wireless electronics and their widespread implementation with increased levels of electromagnetic pollution have led to the quest for developing novel electromagnetic interference (EMI) shielding materials^1–3^. Traditionally metals and metal-based composites have been used effectively for shielding purposes. However, they show several limitations, including, the absorption range within short bandwidth, highly corrosive, and increased weights. This issue was countered by introducing polymer-based composite coatings, which are lightweight, inert, flexible, and low density but exhibited poor absorption bandwidth with narrow frequency regions^4^. In this regards, ceramic ferrites show high promises, since, they exhibit broad absorption frequency in the GHz regions, with high strength, low conductivity, and thus, acts as a magnetic/dielectric filler in with a prime candidate for shielding applications^5^. Specifically, soft ferrites are highly significant in the field of electromagnetic shielding, since, saturation magnetization, coercivity, remanence, anisotropy constants, magnetostriction, and Neel temperature of soft ferrites play a key role in determining its frequency region (i.e., bandwidth)of microwave absorption^6^. Also, the chemical substitution of different size ions assist in changing the magnetization and Neel point, and thus, a wide variety of ferrite compositions can be utilized as high-frequency absorbing materials devices^7^. To date, a wide range of soft magnetic materials has been developed for microwave absorption purpose. To name a few, Co-ferrite^8^, Ni-ferrite, NiZn-ferrite^9^, MnZn-ferrite^10^, MgZn-ferrite^9,11^ and other substituted soft ferrites^12^. Nanocrystalline spinel ferrites exhibit significant changes in the cationic distribution; thus, resulting in improved magnetic properties as compared to their bulk counterpart. For example, ZnFe_2_O_4_ is a normal spinel in its bulk form exhibiting ferromagnetic ordering below 10 K; however, upon forming nanocrystallineZnFe_2_O_4_, it shows magnetically ordered structure with high magnetic moment even at elevated temperatures^13,14^. This phenomenon is primarily attributed to the change in cationic distribution from its normal to the inverse structure.

On the other hand, nanocrystalline NiFe_2_O_4_is an inverse spinel with non-collinear spin structure and lower magnetic moment than its bulk counterpart^14–16^. In this regard, nickel zinc ferrites Ni_1−x_Zn_x_Fe_2_O_4_ (x = 0–1.0), a mixed ferrite whose broadband absorption properties and magnetic properties are strongly dependant on the zinc content, exhibiting antiferromagnetic behavior at x = 0.9 and 1.0 whereas the composition with x = 0.5 shows the best microwave absorption properties^17^. To date, various methods have been used for synthesizing ferrites such high-energy ball mill, co-precipitation method, sol-gel method, auto-combustion method, hydrothermal route, and reverse micelle process. Among those all, sol-gel auto combustion method is a low-cost, rapid, and facile method of synthesizing nanocrystalline ferrite powders with controllable particle size^18,19^.

However, ferrite nanoparticles alone as microwave-absorbing material has its several drawbacks, including, narrow bandwidth, less absorptivity, and less impedance matching. In particular, high magnetic loss with the low impedance matching degrades the microwave absorption properties of ceramic soft-ferrites alone^20^. These problems can be overcome by introducing composites by mixing various carbon allotropes with ceramic ferrites, thus, enhancing the broadband absorption properties while improving strength/weight ratio of composite material. Carbon allotropes like carbon nanotubes^21–23^, carbon nano-fibres^24,25^, and graphene^20^, showed the extraordinary performance of microwave frequency absorption because of their sp^2^ bonded structures exhibit thermally activated carrier hoping associated with defect states, which results in high-efficiency EM wave attenuation^26–28^. Graphene oxide (GO) is atomically thin monolayer graphitic allotropes with highly stable two-dimensional structure with extremely high surface area^29,30^, lightweight, and high dielectric permittivity^28,30^. The combination of magnetic properties of ferrites and high-permittivity GO enhances the microwave absorption properties of ferrite-graphene matrix^31–33^. Furthermore, nanoparticle grafted on graphene membrane can create homogeneous and versatile structures, where a clear dielectric and magnetic interface can form the magneto-dielectric hetero-junction, which could act as a center for microwave attenuation^32^.

In this work, we demonstrate an *in-situ* synthesis of Ni_0.5_Zn_0.5_Fe_2_O_4_ ferrite (NZF)-GO hybrid materials via a sol-gel auto-combustion process. We also demonstrated the controlled particle size formation using the fuel versus oxidizer ratios during reaction while keeping the phase-pure NZF particles grafted on graphene oxide. The morphology of the NZF grafted GO scaffold structure is confirmed using SEM and TEM and HRTEM is used to demonstrate a clear interface formation in between ferrite nanoparticles and GO. Furthermore, we demonstrate that with varying amount of NZF:GO ratios, the hybrid exhibits to optimize the best saturation magnetizations with the lowest permeability. The final goal was to disperse the nanocomposite in a sprayable polymer (polyurethane) and fabricate thin coatings on metal substrates for measuring the microwave absorption properties. The broadband microwave absorption properties of the coated substrates are investigated throughout the broadband range of 4–15 GHz. The samples are found to behave as absorbers in X-band and higher frequencies with absorptivity greater than 90%.TheseNZF-GO scaffold materials are unique, morphologically homogeneous, and scalable, thus, can be widely used in radar absorbing materials, stealth technology, and electromagnetic shielding applications with tuneable bandwidth and varying absorption efficiency.

## Results and Discussions

**Figure 1:**
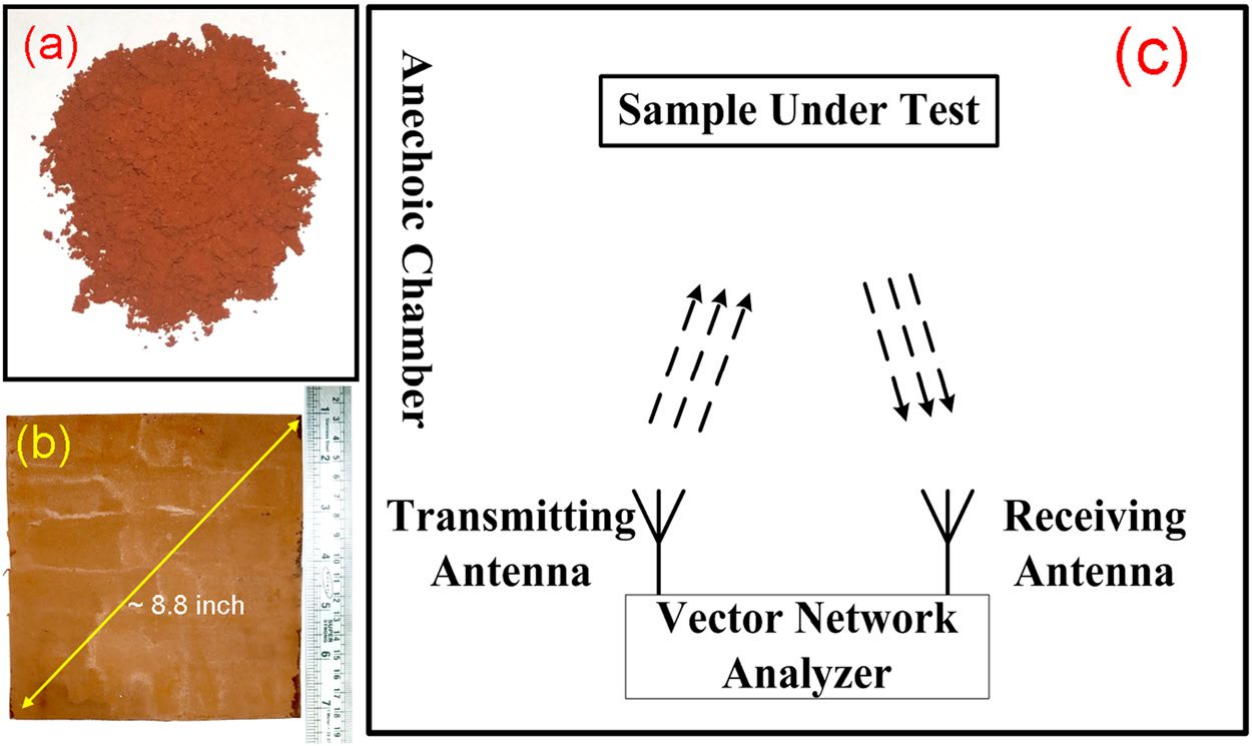
Shows the (**a**) as prepared NZF-GO powder; (**b**) the NZF-GO nanocomposite- TPU coated the aluminum substrate, and (**c**) schematically demonstrate the broadband microwave measurement technique.

We synthesized Nickel Zinc Ferrite of composition Ni_0.5_Zn_0.5_Fe_2_O_4_-GO hybrid scaffold nanocomposite by a sol-gel auto-combustion technique, and we developed a series of NZF-GO nanocomposites with the increasing amount of GO. The amount of GO (concerning the calculated Ni-Zn ferrite yield in w/w ratio)was added initially to the precursor solution during the sol-gel auto-combustion process, which produced a mixture of NZF and GO nanocomposite after completion of the process as shown in Fig. 1a. Three different samples were prepared with the various w/w ratios of NZF and GO varying as 2:1, 1:1, and 1:2. The name of those samples will be addressed as NZF-GO33, NZF-GO50, and NZF-GO66 respectively, throughout the rest of the manuscript where 33, 50, and 66 illustrates the percentages (%) of GO present in the nanocomposite. Figure 1b shows the NZF-GO nanocomposite in polymer coating on a metal substrate (~9-inch diagonal length)as described in the “Materials and Method” section in this manuscript. The as coated metal substrates were used for the free-space broadband measurement in an anechoic chamber, as shown in the schematic in Fig. 1c.

**Figure 2:**
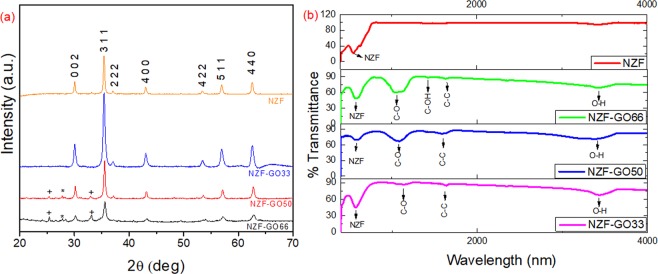
Shows (**a**) the comparative X-ray diffraction analysis and (**b**) Fourier transform infrared spectrograph of pristine NZF and NZF-GO nanocomposites with different ratios of NZF and GO.

The auto-combustion synthesis technique for nanocomposite synthesis is an exothermic process (Section S1, supporting information) where our calculation revealed the amount of heat evolved from the reaction is ~1976 kJ/mol. We believe that the exothermic reaction causes the intercalation of sol particles inside of the GO layers followed by the formation of grafted nanoparticles on GO membrane. Interestingly, the exothermic reaction causes the rapid exfoliation of GO during the reaction, which results in the formation of high-surface-area NZF-GO hybrids with the successive amount of GO in it. Figure 2a shows the X-ray diffraction profile of Ni_0.5_Zn_0.5_Fe_2_O_4_/Graphene Oxide (NZF-GO) hybrid synthesized with different GO content, and those XRD peaks are identified using the standard line profile JCPDS data (No 00-008-0234). The diffraction peaks of all the samples showed the characteristic of a phase- pure Ni-Zn ferrite spinel structure with the highest intensity peak obtained from (311) plane, which is comparable to the pure NZF XRD data (Fig. 2a, the orange curve in the top). The low-angle XRD data of pristine GO is shown in Fig. S2a (Supporting information), which shows the characteristics GO peaks of (001), (002), and (100) at their respective 2θ positions of 9.97°, 24.8°, and 42.52^°^^34^. We found that all those diffraction peaks of NZF-GO hybrids undergo significant peak broadening with the increase in the amount of GO in the hybrids. This may be attributed to the formation of homogeneous particles distributed over the GO membrane, and the degree of agglomeration decreases with increasing the amount of GO content in the hybrid materials. We found that the as-synthesized NZF nanoparticles in GO scaffold for all those samples exhibit cubic structure with Fd-3m space group. It was found that the samples NZF-GO50 and NZF-GO66 with a significant amount of GO content show diffraction peaks of graphite along with spinel phase ferrite indicating the presence of graphitic carbon peaks. We believe that the increase in the GO content provided the more surface area to grow small nanoparticles, thus developed more small nickel zinc ferrite particles, and thus it results in the peak broadening of the XRD peaks for those samples having higher GO content. Since we have used this hybrids and TPU for the preparation of paints and coat metal substrates for broadband absorption measurement, we presented the XRD data of TUP (Fig. S2b, supporting information) to show that the polymer has no contribution in phase development, except play the role as the binder. Figure 2b shows the comparative Fourier transform infrared spectrographs of NZF-GO samples with pure NZF sample and various NZF-GO samples. The occurrence of C-O, C-C, and O-H stretching frequency peaks signify that the presence of graphene oxide in all as-synthesized hybrids^35,36^.

The characteristics peak of NZF is also obtained at the respective positions of ~571 cm^−1^ for NZF-GO33 and found to be redshifted for NZF-GO50 and NZF-GO66 with their respective positions of ~585 cm^−1^ and 589 cm^−1^, respectively, as shown in Figs 2b and S3a (Supporting information)^37,38^. The redshift of the NZF peak with increasing GO content may be due to some interface formation occurred between NZF nanoparticles and GO sheet. On the other hand, the peak position of C-O stretching frequency in NZF-GO33 at 1136 cm^−1^ has been shifted to 1085 cm^−1^ (for NZF-GO50), which has further been shifted to 1045 cm^−1^ for NZF-GO66 and became doublet peak^35^. This may be due to the development of more hetero-interface between NZF and GO in excess quantity. The interactions between NZF nanoparticles and GO have further lead to the formation of heterostructures at the interface, which is elaborated further in the TEM section of this manuscript.

**Figure 3:**
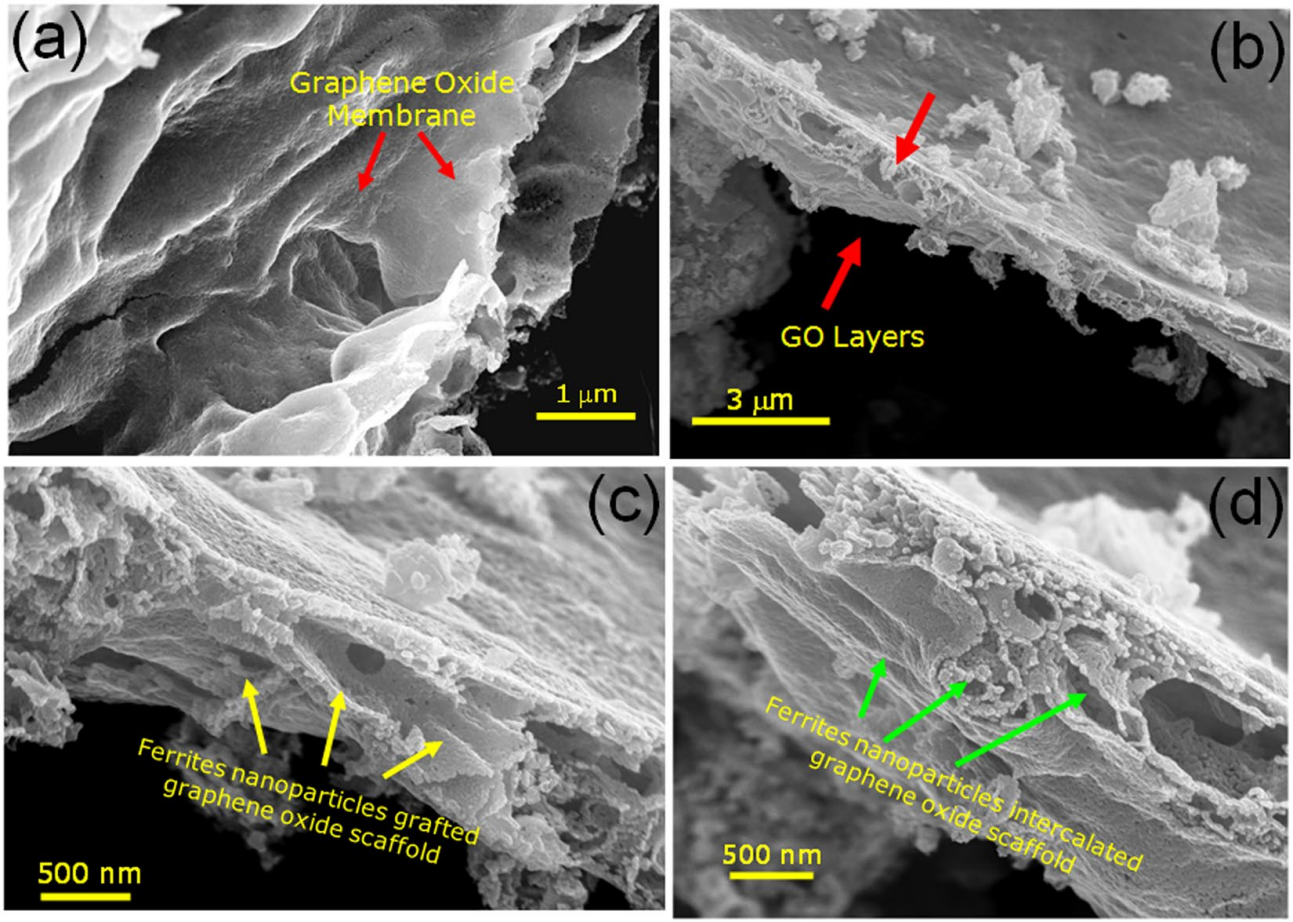
Shows the morphologies of (**a**) GO, and (**b**–**d**) various NZF nanoparticles grafted GO nanocomposites under different magnifications.

Figure 3 shows the SEM images of as-synthesized GO and NZF-GO hybrid with a significantly different morphological structure where small NZF nanoparticles were found to be grafted on graphene oxide layers. Figure 3a demonstrates as-synthesized pristine GO with large flake, typically crumpled, and flaky structure. Figure 3b shows NZF-GO66 hybrid morphologies where it is observed that NZF nanoparticles are grafted on the layered structure of GO. Also, it can be seen that the GO layers became highly exfoliated during the gel-combustion synthesis process, thus, created a large-surface-area NZF coated homogeneous GO nano-scaffold as shown in Fig. 3c,d. Figure 3c,d depict the high-magnification image illustrating the hybrid framework (high magnification images of Fig. 3b) of NZF grafted GO layers of various regions of the image shown in Fig. 3b. From Fig. 3c,d, it is observed that some of the tiny NZF nanoparticles were intercalated through the GO layers as well during the *in-situ* formation of nanocomposites. Since the size of NZF nanoparticles was found to be in the range of 15–20 nm, we will discuss the size and size distribution of NZF particles in the next part of the TEM section.

**Figure 4:**
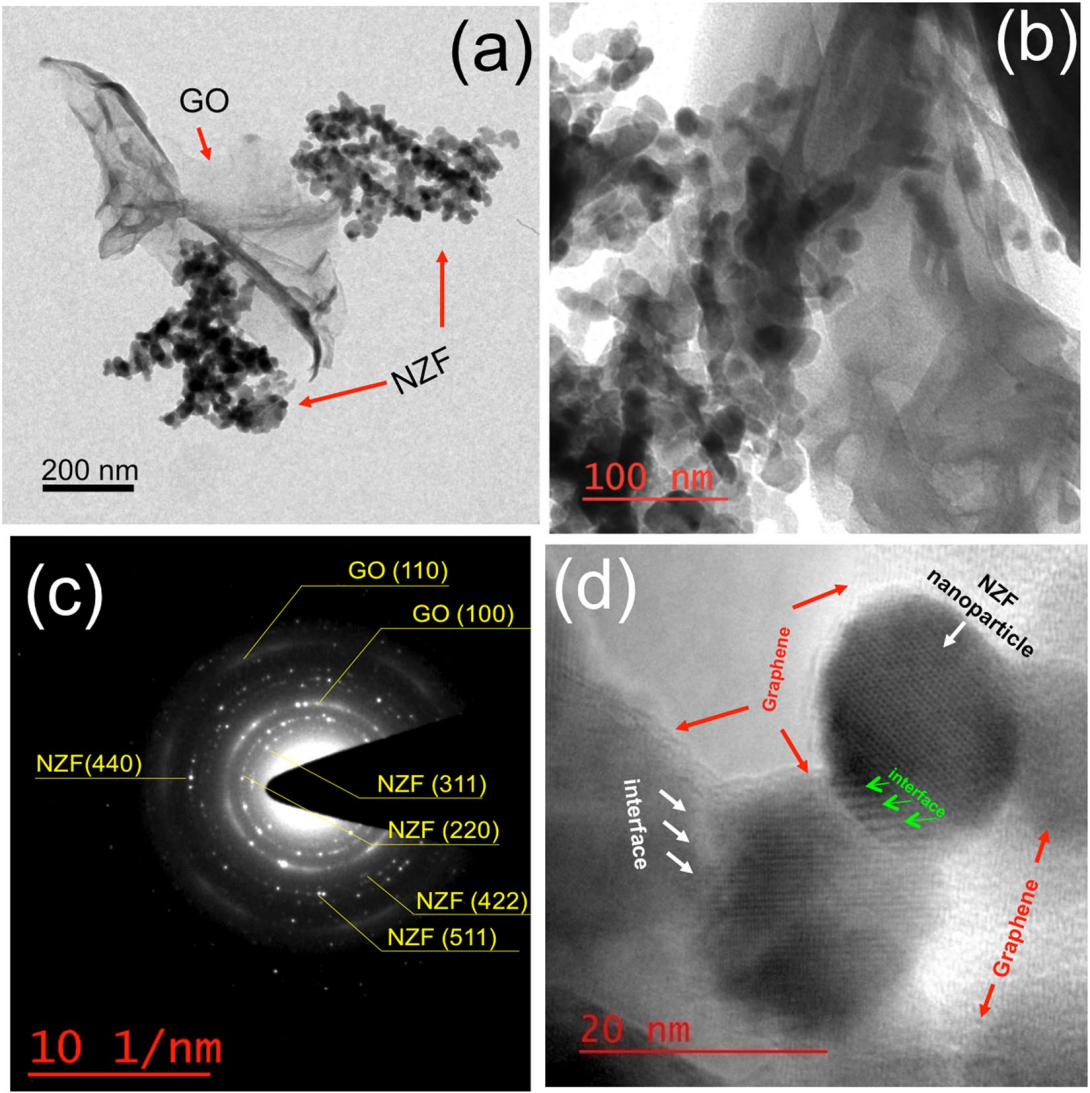
Illustrate TEM of (**a**) NZF-GO33 sample and (**b**) NZF-GO66 sample. NZF-GO66 sample shows that NZP particles are anchored on the GO scaffold and forms nanocomposite structure; (**c**) SAED pattern showing the nanocomposite structure of (**b**), where both the lattice planes of GO and NZF are clearly visible; (**d**) HRTEM picture showing the heterogeneous interface formation between NZF particle and GO for the NZF-GO66 sample.

Figure 4 shows the TEM/HRTEM pictures of the NZF-GO samples with different ratios of NZF and GO. Fig. S4a (Supporting Information) shows the TEM image of as-synthesized pristine GO flake of size ~500–800 nm. A large-scale nanocomposite structure under low magnification TEM is shown in Fig. S4b (supporting information) depicting the constitution of structurally homogeneous hybrid scaffolds for NZF-GO sample. Figure 4 as hows the transmission electron micrograph of NZF-GO33 depicting the agglomerated NZF over the GO sheet due to the cohesive force of excess NZF. On the other hand, homogeneously dispersed NZF over GO sheet is observed for NZF-GO66 (Fig. 4b) thereby creates large interfacial contact between NZF nanoparticles and GO. The nanocomposite was further verified using a SAED pattern, as shown in Fig. 4c. In the SAED pattern, it was observed that both the NZF nanoparticles and GO structures are co-existed in the scaffold as evidenced with the characteristics planes of GO (110) and (100) and the characteristics planes of NZF of (311), (220), (422), (440) and (511).

Interestingly, the SAED pattern exhibits some scattered bright dots, which manifests the formation of dispersed nanoparticles with highly crystalline structures on GO membrane. Figure 4d shows the HRTEM image of interfaces formed between NZF particles and GO. As could be seen from Fig. 4d, those interfaces are hetero-interfaces with significantly different structures in comparison to the structure of either GO or NZF. We believe that the nanoparticle is implanted through the GO interlayer, which occurred during the sol-gel synthesis process while exothermic reaction (as described in the Section S1, supporting information) causes the formation of a bond between GO membrane and NZF nanoparticles. Since the particle size of the NZF nanoparticles are ~17 ± 4 nm, it is quite obvious that the well-connected GO layers supposed to be separated away and forms a nanocomposite with the high-surface-area. It could also be presumed that the hetero-junction interfaces between NZF and GO act as a magneto-dielectric junction between a magnetic nanoparticles and a dielectric layer, hence, it may be turned into a larger boundary array in NZF coated GO membrane^39,40^. We believe that those unique hetero-junction interfaces with distinct characteristics like a larger magneto-dielectric layer array solely acts as GHz frequency absorber with wideband absorption characteristics as discussed in the later sections.

**Figure 5:**
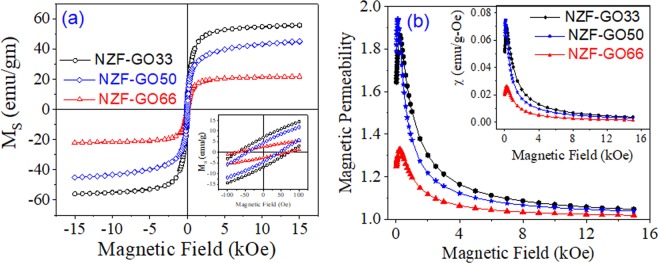
Shows the (**a**) saturation magnetization (M_S_) vs. Magnetic field, while the inset shows the characteristics of coercivity of NZF-GO hybrids; (**b**) demonstrates the magnetic permeability vs. magnetic field curve and magnetic susceptibility vs. the magnetic field (inset) of NZF-GO hybrids with increasing GO content.

Figure 5 shows the magnetic characterizations of the NZF-GO hybrids with various GO contents. Figure 5a depicts the magnetization (MS) vs. applied field (H) plot of all NZF-GO hybrid materials where the slim hysteresis curves signify a typical soft-ferrimagnetic characteristic for all the samples. It is seen from Fig. 5a that there are significant differences in saturation magnetization (M_S_) values of the various NZF-GO hybrids. It could also be observed that with increasing GO content, the *M*_*S*_ decreases, which is due to the paramagnetic nature of GO^41^. As GO acts as a membrane of the hybrids, it is quite obvious to degrade the magnetic moment of the composite with higher GO content. However, there are no significant changes found in the coercivities of all those three hybrids (inset of Fig. 5a); therefore, this validate that all NZF-GO hybrids exhibit soft-ferrimagnetic characteristics. Figure 5b demonstrates the characteristics of magnetic permeability (µ) vs. applied magnetic field plot of all three different hybrid samples with a varied amount of GO content. Figure 5b inset delineates the magnetic susceptibility (χ) of those hybrids and found that NZF-GO66 (i.e., the highest GO content nanocomposite) exhibits the lowest µ value as well as the lowest χ value. In general, the permeability of a material can be defined as its ability to support the formation of a magnetic field within itself and it allow passing through the magnetic lines of force. Therefore, it could be observed from Fig. 5b that with the increasing amount of GO content, all hybrid materials showed strong resistance towards magnetic lines of force to pass through it in the low field region. This could be due to the effect of the increasing amount of NZF-GO heterostructural magneto-dielectric interface, which may be diamagnetic. Overall, it indicates that any incident magnetic field will either be absorbed or get reflected the most from the NSF-GO66 sample in comparison with the other two samples. Furthermore, the NZF-GO66 should show the maximum broadband-microwave absorptivity of the incident radiation in the GHz frequency range, and our broadband measurement data are quite aligned with these magnetic characterizations data as elaborated in the next section.

**Figure 6:**
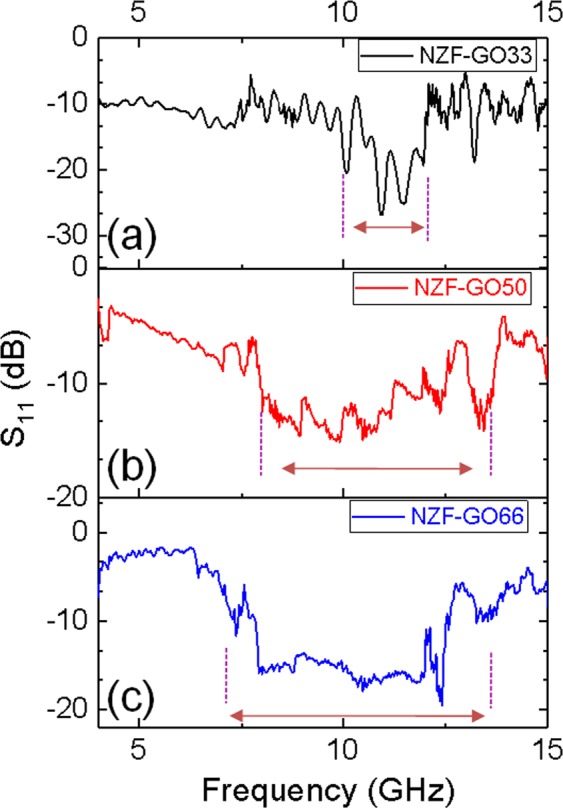
Shows the comparative reflectivity (S11) data of various NZF-GO nanocomposite samples with a varied amount of GO content. The composite was dispersed in polyurethane to form paint and coated over Al substrates for measuring their performance as GHz absorber coating.

The as-prepared NZF-GO hybrids were dispersed in polyurethane polymer (as discussed in detail in the Materials and Method section) and coated on the aluminum substrates of thickness ~100–120 μm. The thickness of the aluminum sheet is chosen greater than the skin depth at the frequencies under consideration so that the incident electromagnetic wave to the sample should not undergo any transmission through it. The standard free space measurement has been performed to measure the reflectivity of the sample under test within the frequency range 4 to 15 GHz, as shown in the schematic in Fig. 1c. Initially, the reflection coefficient has been measured by placing the aluminum substrate alone, which acts as an ideal reflector followed by the polymer coated aluminum substrate. Next, the reflection coefficient has been measured from the nanocomposite coated aluminum substrates under identical conditions. Step-By-Step measurement techniques are described in Fig. S1 (supporting information). Figure S5 (supporting information) shows the as collected reflection data (i.e., S_11_) from the various sample surfaces, which includes the data from an aluminum substrate, aluminum/polymer, and aluminum/polymer/various NZF-GO samples. Thus, the difference between the three reflections gives rise to the actual reflection coefficient from the various NZF-GO samples as depicted in Fig. 6 and as well as from the polymer (Fig. S5, supporting information). It is also observed that the polymer used for the coating possesses minimum reflection from its surface as observed from Fig. S4.

It is observed from Fig. 6 that broadband reflection minima have been achieved in X-band, particularly with the increase of the percentage of GO within the NZF-GO nanocomposites as seen in Table 1. The bandwidth has been significantly enhanced with the increase of the GO percentage within the mixture. Owing to the insulating properties of GO, the incident electromagnetic energy gets trapped within the sprayed sample and the aluminum metal placed at the backside.

**Table 1:** Demonstrates the comparison of −10 dB bandwidths of NZF-GO composites with increasing GO content.

Sample Types	Range of −10 dB bandwidth (GHz)	Bandwidth (GHz)
*NZF-GO33*	10.40–12.06	2.66
*NZF-GO50*	7.96–12.48	4.52
*NZF-GO66*	7.84–13.80	5.96

The absorptivity of the structure can be computed from Eq. (1) where *S*_11_ and *S*_21_ are the reflection coefficients and transmission coefficients, respectively, from the sample while A is the absorptivity.

1 $$A=1-{|{S}_{11}|}^{2}-|{S}_{21}{|}^{2}$$

Now, since the sample is completely metal-backed (aluminum sheet), there is no transmission through the sample, implying S_21_ = 0. Hence, the absorptivity of the sample is calculated using Eq. (2)^42^.

2 $$A=1-{|{S}_{11}|}^{2}$$

**Figure 7:**
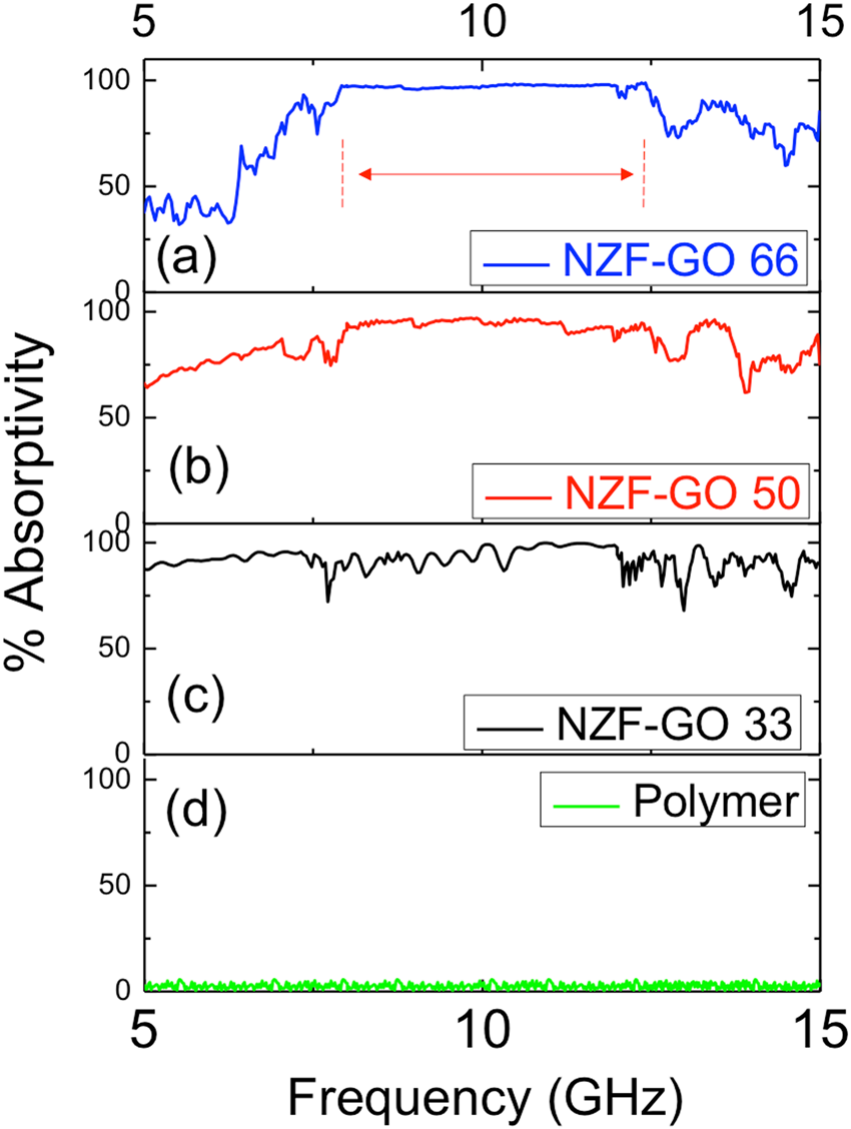
Demonstrates comparative broadband absorption data of various NZF-GO samples with an increasing amount of GO content.

To check the reliability, we have performed each measurement for all the coatings three times under the same condition, however with varying orientations and the data as shown in Fig. S6 (supporting information). It is observed that all samples exhibit nearly identical reflection losses in all the trials as evident from Fig. S6 (supporting information). Consequently, the reported bandwidths obtained using all the nanocomposite samples remain almost invariant (Table 1). It is further observed from Table 1 that absorptions primarily occur in X-band and higher frequency band with the increase of GO content in the original NZF sample. The comparative absorptivity plots for all the NZF-GO samples are illustrated in Fig. 7a–c, which demonstrates different wideband absorptivity for each sample. It is also seen from Fig. 7a that NZF-GO66 exhibits broad and wideband absorptivity >90% within the range of 7.84 GHz to 13.80 GHz. The fractional bandwidth ~55% has been achieved with respect to 10.8 GHz (the center frequency of the absorption band) in case of NZF-GO66 sample while NZF-GO33 and NZF-GO50 exhibit fractional bandwidth of 23.7% and 44.2%, respectively. Interestingly, the fractional bandwidth is found to be increased for nanocomposites with increasing GO/NZF ratio, which has added advantages of making the nanocomposites lightweight and durable. It is again observed that practically no absorption takes place in polymer coating as depicted in Fig. 7d.

The absorption bandwidth and thickness of the thin coating sample made with NZF-GO66 dispersed in TPU polymer was compared with the available data reported in recent literature, and comparative results are demonstrated in Table 2. From Table 2, it is found that our material provides improved absorption bandwidth, maintaining better compactness in compared with the recently reported data.

**Table 2:** Illustrates the comparative data published in recent literature and our experimental results obtained in this work.

Literature	Bandwidth	Thickness
Shu *et al*.^46^	3.2 GHz	2 mm = λ/15
Yang *et al*.^47^	7.8–12.6 GHz	5 mm = λ/6
Liu *et al*.^20^	4.8 GHz	3 mm = λ/10
Liu *et al*.^48^	6 GHz	2 mm = λ/11
Zhang *et al*.^31^	4 GHz	1.4 mm = λ/20
Proposed NZF-GO66 sample	5.96 GHz (=7.84–13.8 GHz)	1 mm = λ/30

**Figure 8:**
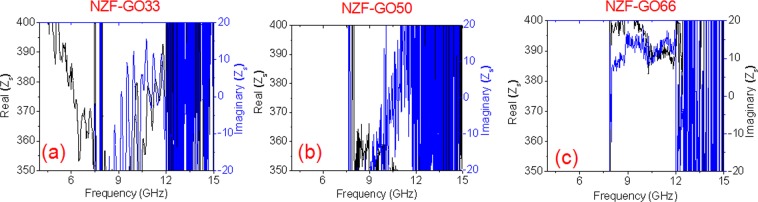
Shows surface impedance of various nanocomposites with a successive increase of GO content (**a**) NZF-GO33 (**b**) NZF-GO50 and (**c**) NZF-GO66.

Furthermore, to analyze the absorption phenomena, the surface impedance of the NZF-GO nanocomposites have been calculated. The real parts and imaginary parts of surface impedances are illustrated in Fig. 8. For all these NZF-GO nanocomposites, the surface impedance was calculated as per Eq. 1. The as-measured surface impedance of the samples should match with the input impedance of a transmission line with characteristic impedance Z_0_^43^. The surface impedance was calculated from the measured reflection coefficient using Eq. 1. Here, the surface impedance *Z*_S_ was calculated from the measured reflection coefficient *S*_11_ and free space impedance *Z*_0_.

3 $${Z}_{S}={Z}_{0}\frac{1+{S}_{11}}{1-{S}_{11}}$$

The change in real components and imaginary components of surface impedances of three distinct nanocomposites are presented in Fig. 8, which demonstrates the complete absorption of incident electromagnetic wave, when the as-calculated surface impedance is accurately matching to the characteristic free-space impedance (i.e., ~377 Ω). It is observed that the real part and imaginary part of the surface impedance are close to 377 Ω and 0 Ω, respectively, over the desired band of absorption (i.e., X-band) for the samples as listed in Table 1. However, the impedance values (i.e., real and imaginary parts) are found to be almost 377 Ω and 0Ω for NZF-GO66 sample in comparison with the other two samples. For further elucidation one to one comparative plots of RF absorption versus frequency and impedance plots versus frequency is demonstrated in Fig. S7 (supporting information).

In summary, we found that NZF-GO66 shows the best microwave absorption properties along with the enhancement (almost ~55%) in fractional bandwidth throughout the X-band. We know that nanoscale materials are very easily polarized under an electromagnetic field; thus, it can be assumed that each nanoparticle act as an electric dipole in the electromagnetic field, which is again very susceptible to space charge polarization by trapping space charges and enhancing the microwave properties. Here we observed that with increasing GO content in the composite, the dispersibility of the nanoparticles is enhanced, which results in the formation of smaller particle size (i.e., higher surface area) uniformly distributed on the GO layers. Moreover, it can be assumed that by enhancement in dispersibility, it increases the formation of heterostructure interfaces (i.e., magneto-dielectric interfaces as discussed in TEM/HRTEM section and magnetic data analysis section) between the NZF nanoparticles and GO, which act as the center of microwave absorption. This may be due to the formation of interfacial magnetic dipoles, which leads to the formation of more active interfaces, are one of the key characteristics of absorbing electromagnetic waves. The larger number of heterostructural interfaces results in higher surface area as well as highest compactness, which improves the microwave absorption along with enhancement in fractional bandwidth. Thus, the larger the magneto-dielectric interfacial area, lower penetration occurred by electromagnetic waves. In brief, we have demonstrated the efficacy of the ferrite-GO nanocomposites towards the broadband-microwave frequency absorption by embedding the ferrite nanoparticle in GO membrane while nanocomposite dispersed polymer paint showed sophisticated processing and easy application as a lightweight coating on metal surfaces.

## Conclusions

In conclusion, we have demonstrated the successful synthesis of *in-situ* Ni_0.5_Zn_0.5_Fe_2_O_4_ ferrite nanoparticle grafted-graphene oxide hybrid scaffold as broadband microwave absorber material. We obtained phase pure NZF particles with an average particle size of ~17 nm± 4 nm, which was found to be homogeneously coated on to GO layers. The scaffold exhibits NZF nanoparticles grafted on GO, which form during the *in-situ* gel combustion process. Various heterogeneous interfaces are formed between NZF and GO layers with distinct lattice structures and bonding. The formation of interfacial bonding occurred between NZF and GO during the sol-gel auto-combustion process as the reaction is highly exothermic. The magnetic data show that the saturation magnetizations, as well as magnetic permeability decreases for the hybrid structure with an increasing amount of GO as GO, are diamagnetic. Furthermore, the nanocomposite coatings showed excellent broadband absorption properties throughout the microwave frequency range (8–12 GHz) with absorptivity greater than 90%. More specifically, with increasing GO content, the bandwidth of RF absorptions for individual nanocomposite became widen while the absorptivity of corresponding nanocomposites is found to be significantly improved. The real and imaginary parts of the impedance of the hybrid nanocomposite coatings are obtained as 377 Ω and 0 Ω, respectively, over the entire region of the frequency band. This further indicates that the lowest reflections from the hybrid-coating over the frequency band of interest, thus, contributing towards broadband absorption in the range of 8–12 GHz. We obtained the improvement in dispersibility of nanoparticles with increasing GO content, which causes the formation of a large number of magneto-dielectric hetero-junction interfaces. Thus, the interfaces cause trapping of space-charge along with magnetic dipoles, which effectively enhance the absorption bandwidth as well as improvement in absorptivity. These types of graphene oxide hybrid framework based hybrid-nanocomposite coatings on metal will find immense possibilities for applications as radar absorbing materials, stealth technology, electromagnetic shielding, and the radiated EMI management in onboard spacecraft and safeguarding medical devices form electromagnetic radiations.

## Materials and Method

### Preparation of graphene oxide

Graphene oxide was synthesized via oxidation of graphite flakes obtained from Merckmillipore, Germany (~150 μm flakes) via improved method reported elsewhere^44^.

### Preparation of Ni_0.5_Zn_0.5_Fe_2_O_4_-GO scaffold

Ni_0.5_Zn_0.5_Fe_2_O_4_was synthesized by gel combustion method. Graphene oxide was added *in situ* during the process. The chemical reagents used for synthesis were Fe(NO_3_)_3_.9H_2_O, Ni(NO_3_)_2_.6 H_2_O, Zn(NO_3_)_2_.6 H_2_O, de-ionized water (Millipore), citric acid anhydrous and ammonia solution (All purchased from Merckmillipore, Germany and Sigma-Aldrich, India). In a typical experiment, metal nitrates in the required molar ratio were dissolute in the minimum amount of distilled water. A stoichiometric amount of citric acid was used as fuel for the experiment. A small amount of ammonia solution was added to the solution to adjust the pH value to about 6. After stirring the solution at room temperature for 30 minutes, graphene oxide was added to the solution and ultra-sonicated for 15 minutes. The ultra-sonicated solutions containing GO were evaporated at 100 °C on a hot plate while continuously stirring to transform them into dark brown viscous xerogel. By further raising the temperature to 220 °C the gel was ignited in the air with the evolution of the large volume of gases and produced a dark-brown mass was obtained. The as-obtained powder from the gel-combustion technique was calcined at 600 °C for 2 hours, as shown in Fig. 1a.

### X-Ray diffraction analysis

The phase analysis of all the NZG-GO powders was performed using X-ray diffraction (XRD) analysis technique in an XRD instrument (RigakuSmartLab, RIGAKU Corporation, Japan). All the diffraction peaks were analyzed using standard line profile JCPDS data (No. 00-008-0234).

### Fourier transform infrared spectroscopy

Fourier transform infrared spectroscopies were carried out using FTIR spectrometer (THERMO Electron Scientific, Instruments LLC, Model No: Nicolet iS5) from the ranges of 400 cm^−1^ to 4000 cm^−1^ with the resolution of 4 cm^−1^.

### Scanning electron microscopy

The morphologies of the NZF-GO scaffold materials are characterized using a scanning electron microscope (SEM) equipped with energy dispersive spectroscopy (EDS) (NOVA FESEM 450, FEI USA).

### Transmission electron microscopy/high-resolution transmission electron microscopy

All the nzf-GO scaffold nanocomposites were characterized in a transmission electron microscope (TEM) (TECNAI G2 20 TWIN, FEI, USA) where the as-synthesized nanocomposite samples were dispersed in solvent and drop cast on a lacy carbon coated TEM grid.

### Measurement of magnetic properties

The magnetic properties of all the NZF-GO samples were characterized using a vibrating sample magnetometer (VSM) (Model: EZ9, Microsense, USA). All the NZF-GO samples were filled in acrylic cups followed by fixing it to quartz rods for mounting on the VSM. Before the magnetic measurement, the samples were A.C. demagnetized with a demagnetization factor of 0.9 for removing any stray magnetization from the samples. Following that the M-H measurements were performed. The same measurement conditions were used for the respective background corrections.

### Fabrication of nanocomposite-polymer coating

For the nanocomposite coating fabrication, we have purchased thermoplast polyurethane (TPU) (Bayer Material Science AG: polyester based TPU (Desmopan 385S)) with the shore hardness of 86A. This polymer was equivalent materials used in conventional paints for different coatings^45^. We took TPU and different NZF-GO scaffolds in 1:1 weight ratio for nanocomposite fabrication where TPU was dissolved in dimethylformamide (DMF) by stirring it at 100 °C followed by the addition NZF-GO scaffold powders. The solution was ultra-sonicated under high power probe sonicator for one hour followed by transferring those solutions on a hot plate (100 °C) under constant stirring. The NZF-GO scaffold dispersed TPU solution was coated onto Al substrates of dimension 8.5 inches (diagonal length) using a simple hand-brushing. The photographic image of the as-coated substrate is shown in Fig. 1b.

### Measurement of broadband microwave properties

The microwave absorptivity of the as-fabricated NZF-GO nanocomposite-TPU coating on Al-substrates was measured inside of an anechoic chamber. We have used three different pairs of horn antennas (viz. C-band antenna, X-band antenna, and Ku-band antenna) for measuring the reflection from the as-fabricated coating on Al-substrate. In the measurement set-up, from each pair of horn antennas, one was used as a transmitter to send off the electromagnetic wave on to the sample whereas the other antenna was used to receive the reflection from the sample. All the horn antennas were connected to the vector network analyzer (VNA) (Anritsu MS 2037, Japan) during the entire measurements. The entire broadband absorption measurement techniques are shown schematically in Fig. S1 (Supporting information).

## Supplementary information

Supporting Information
