# Supplementary material for: Graphene oxide-ferrite hybrid framework as enhanced broadband absorption in gigahertz frequencies
Source: Sci Rep. 2019 Aug 20;9:12111. doi: 10.1038/s41598-019-48487-5 (PMC6702174; doi:10.1038/s41598-019-48487-5)
Supplement: Supplementary file 1 — Supporting Information [file 41598_2019_48487_MOESM1_ESM.docx]

Supporting Information

**Graphene oxide-ferrite hybrid framework as enhanced broadband absorption in gigahertz frequencies**

Rajarshi Bhattacharyya^1,5^, Om Prakash^2^, Somnath Roy ^3^, AkhilendraPratap Singh^4^, Tapas Kumar Bhattacharyya^5^, Pralay Maiti^2^, Somak Bhattacharyya^4^, and Santanu Das^1^*

^1^ Department of Ceramic Engineering, Indian Institute of Technology (Banaras Hindu University), Varanasi Uttar Pradesh 221005 INDIA

^2^ School of Materials Science and Technology, Indian Institute of Technology (Banaras Hindu University), Varanasi Uttar Pradesh 221005 INDIA

^3^Department of Physics, Institute of Science, Banaras Hindu University, Varanasi Uttar Pradesh 221005, INDIA

^4^Department of Electronics Engineering, Indian Institute of Technology (Banaras Hindu University), Varanasi Uttar Pradesh 221005, INDIA

^5^Department of Ceramic Technology, Govt. College of Engineering and Ceramic Technology, Kolkata, West Bengal 700010 INDIA

*E-mail:, Fax: +91-542-2368428

*Corresponding author. Tel: +91-9918910417. E-mail: santanudas.cer@iitbhu.ac.in (Santanu Das)

**Section S1: Calculations involved with the gel combustion synthesis**

Ni_0.5_Zn_0.5_Fe_2_O_4_ was synthesized by gel combustion method where citric acid was used as fuel.

The general reaction involving the synthesis was:

**0.5**[Ni(NO_3_)_2._6H_2_O] +**0.5**[Zn(NO_3_)_2._6H_2_O] +2 [Fe(NO_3_)_3._9H_2_O] +**2.22**C_6_H_8_O_7_ = Ni_0.5_Zn_0.5_Fe_2_O_4_+**4**N_2_+**13.32**CO_2_+**32.88**H_2_O

Where, the molar C/N ratio is 0.27 whenstoichiometric amount of fuel has been used for the synthesis.

The heat of reaction (Q) is given by the difference in the standard enthalpies of formation of the combustion products and reactants.

Q = Δ H _j_°=$\sum_{j} n_{j}A_{j}$Δ _f_ H _j_°-Σ_i_n_i_Δ_f_H_i_°……………. (1)

Where, i and j specify reactants and products respectively and n _i_and n _j_ are the amount of reactants and products. The standard enthalpy of formation of the reactants and products are shown in Table S1.

The adiabatic combustion temperature (T_ad_) is defined as the temperature to which the reaction products are heated using the complete energy under adiabatic conditions and can be solved from the following equation,

T_ad_ = T_o_+ Q/C_p………_(2)

Where C_p_= $\sum_{j} n_{j}A_{j}$ is the average specific heat capacities of the products at room temperature.

The heat of reaction, adiabatic flame temperature (from eq. 5) and moles of gas evolved are listed in Table S2.

Table S1. Enthalpy of formation and specific heat for the combustion synthesis of Ni-Zn ferrite ^1, 2^.

| COMPOUND | ΔH_f_°(kJ/mol) | C_p_(J/mol.K) |
| --- | --- | --- |
| Ni(NO_3_)_2_.6H_2_O (c) | -2211.7 |  |
| Zn(NO_3_)_2_.6H_2_O (c) | -2306.64 |  |
| Fe(NO_3_)_3_.9H_2_O (c) | -3285.3 |  |
| C_6_H_8_O_7_ (c) | -1543.8 |  |
| Ni_0.5_Zn_0.5_Fe_2_O_4_(c) ^a^ | -1041 | 148 |
| CO_2_ (g) | -393.5 | 43 + 0.001 T (K) |
| H_2_O (g) | -241.8 | 30 + 0.015 T (K) |
| O_2_ (g) | 0 | 25 + 0.015 T (K) |
| N_2_ (g) | 0 | 27 + 0.004 T (K) |

^a^ Due to lack of thermodynamic data of Ni-Zn ferrite the average enthalphy and specific heat of Fe_3_O_4_ and CuFe_2_O_4_ were used since these compounds are isostructureand the radii of these divalent ions (Fe^2+^,Cu^2+^,Zn^2+^,Ni^2+^) are nearly the same ^3,4, 5^.

Table S2: Heat of reaction, adiabatic flame temperature and moles of gases evolved for a specified citrate nitrate ratio of nickel zinc ferrite.

| C/N mole ratio | Q(kJ/mol) | T_ad_ (K) | Moles of gases evolved |
| --- | --- | --- | --- |
| 0.27 | 1975.8 | 1386.49 | 50.2 |

**Section S2: Procedures for broadband microwave absorption measurement**

Figure S1: Shows the step-by-step measurement procedure of reflection coefficients for the different nanocomposite samplesusing free space technique.

**Section S3: X-ray diffraction of thermoplast polyurethane (TPU)**

Figure S2: Shows the (a) low-angle XRD pattern of pristine GO with its characteristics peaks; (b) XRD pattern of thermoplast polyurethane (TPU) used for nanocomposite coating illustrating the amorphous nature of the polymer. Thus, we believe that polymer acts as binder for the coating formation and indeed, polymer has no role in contribution of any new phases or so in those as synthesized nanocomposites.

**Section S4: FTIR**

**Figure S3:**Shows the comparative FTIR plots illustrating (a) NZF stretching vibration peak at 571 nm (for NZF-GO 33), which was found to be shifted to 591 nm with increasing GO content; (b) demonstrated the C-O stretching vibration peak of GO and the intensity of the C-O bond was found to be increased with increasing GO content. For composition NZF-GO 66, it is clearly seen from the plot that C-O peak get bifurcated and forms two peaks, which further demonstrated that some sort of interaction occurred between NZF nanoparticles and GO.

**S5: TEM images**

**Figure S4:** Shows the transmission electron micrograph of (a) as synthesized pristine GO flake;(b) NZF-GO 66scaffold structure.

**S6: Microwave Absorption**

**Figure S5:** Demonstrate the as measured S_11_ vs frequency data for all the NZF-GO samples

**Figure S6:** Shows the trial runs of measured reflection coefficients for the different nanocomposite samplesusing free space technique.

**Figure S7:** Demonstrate the one to one figures of S_11_ vs. frequency and the impedance vs. frequency for the different NZF-GO nanocomposites.

**References:**

1. Varma, A.; Mukasyan, A. S.; Rogachev, A. S.; Manukyan, K. V., Solution Combustion Synthesis of Nanoscale Materials. *Chemical Reviews* **2016,***116* (23), 14493-14586.

2. Wagman, D. D.; Evans, W. H.; Parker, V. B.; Schumm, R. H.; Halow, I.; Bailey, S. M.; Churney, K. L.; Nuttall, R. L., The NBS Tables of Chemical Thermodynamic Properties. *J. Phys. Chem. Ref. Data* **1982,***11* (2), 1-407.

3. Hwang, C. C.; Tsai, J. S.; Huang, T. H.; Peng, C. H.; Chen, S. Y., Combustion synthesis of Ni-Zn ferrite powder - influence of oxygen balance value. *Journal of Solid State Chemistry* **2005,***178* (1), 382-389.

4. R.V. Mangalaraja, S. Ananthakmar, P. Manohar, F.D. Gnanam, M. Awano, Mater. Sci. Eng. A 367 (2004) 301–305.

5. Y.M. Chiang, D.P. Brinie III, W.D. Kingery, Physical Ceramics: Principles for Ceramic Science and Engineering, Wiley, New York, NY, 1997, p. 16.
